# Effectiveness of maternal COVID-19 vaccination varies by gestational timing: results from a claims-based cohort study, 2020–2022

**DOI:** 10.21203/rs.3.rs-9772693/v1

**Published:** 2026-06-01

**Authors:** Stacey L Rowe, Matthew M Coates, Sheena G Sullivan, Julia J Koerber, Flor M Muñoz, Onyebuchi A Arah, Annette K Regan

**Affiliations:** Kaiser Permanente; University of California, Los Angeles (UCLA); Monash University; University of California, Los Angeles (UCLA); Baylor College of Medicine; University of California, Los Angeles (UCLA); Kaiser Permanente

**Keywords:** COVID-19, SARS-CoV-2, pregnancy, vaccine, trimester

## Abstract

We evaluated COVID-19 vaccine effectiveness (VE) during pregnancy by dose and trimester in a claims-based cohort of 414,909 pregnant women from 25 December 2020 to 16 September 2022. Vaccination was modeled as a time-varying exposure using weighted Cox proportional hazards models to estimate VE against medically-attended COVID-19 and COVID-19–related hospitalization. Overall, 58,975 (14.2%) women received ≥ 1 dose during pregnancy. We identified 39,202 COVID-19 cases, including 1,130 hospitalizations, with most outcomes (> 93%) occurring during unvaccinated person-time. VE against medically-attended COVID-19 was low-to-moderate across trimesters, with the highest estimates after late second trimester vaccination (36.3%, 95% CI: 26.9–44.4) or ≥ 2 doses (40.7%, 95% CI: 26.3–52.3). VE against hospitalization was higher, albeit with wider confidence intervals (late second trimester, 73.1%, 95% CI: 13.3–91.6). COVID-19 vaccination during pregnancy provided meaningful maternal protection, particularly against severe disease, with some indications that protection may be greatest when given during later gestational periods.

## BACKGROUND

Severe acute respiratory syndrome coronavirus 2 (SARS-CoV-2) infection during pregnancy can cause severe COVID-19, and – compared to non-pregnant women of reproductive age – increases the risk of hospitalization, intensive care unit admission, mechanical ventilation, and death.(1) SARS-CoV-2 infection during pregnancy also increases the risk of pregnancy complications and adverse birth outcomes such as hypertensive disorders of pregnancy, preterm birth, and neonatal intensive care unit admission.(2) Although COVID-19 incidence has reduced considerably since the pandemic was no longer classified as a public health emergency of international concern, these known risks persist for women infected with SARS-CoV-2 during pregnancy.(3)

Vaccination against COVID-19 during pregnancy is safe and effective (4) and, despite evolving national guidelines, continues to be recommended for every pregnancy by the World Health Organization (5) and the American College of Obstetricians and Gynecologists (ACOG).(6) COVID-19 vaccination during pregnancy has the dual benefit of protecting both the pregnant person (4) and their infant (6–8) through transplacental passage of antibodies.(9) Although the vaccine is recommended to be given at any gestational age during pregnancy, the effectiveness of vaccination in preventing maternal disease according to the gestational timing of administration is not well understood. Previous studies have examined the trimester of vaccination on neutralizing antibody activity in maternal and cord plasma samples, demonstrating higher concentrations when the vaccine is administered later in gestation;(10) however, there is comparatively limited direct evidence of the effectiveness of vaccines by trimester. To evaluate whether vaccine effectiveness (VE) varies by gestational timing of vaccine administration, we estimated the effectiveness of COVID-19 vaccination during pregnancy by the number of doses and trimester of vaccination against: (1) any medically-attended outpatient or inpatient maternal COVID-19, and (2) maternal COVID-19 hospitalization.

## METHODS

### Study design, data sources, setting, and participants

We conducted a retrospective cohort study of women in the United States (US) who were pregnant between 25 December 2020 (i.e. two weeks after the availability of the COVID-19 vaccine in the US) and 16 September 2022 (i.e. two weeks before the end of data availability) using the Merative^™^ Marketscan^®^ Commercial Database and the Merative^™^ Marketscan^®^ Multi-state Medicaid claims database. These databases capture de-identified, patient-level data from privately insured employees (and their dependents) and Medicaid enrollees, respectively.(11) Available data included inpatient and outpatient medical claims, outpatient pharmaceutical claims, and facility-level data. Commercial insurance claims additionally provided information on billed laboratory testing and results. These data were used to identify pregnancy-related procedures and tests, laboratory and prescription drug claims, and information on vaccines recorded by physicians, employers, insurance companies, mail-order prescriptions, and specialty pharmacies (see Supplementary Material).

Study participants were women aged 18–49 years, inclusive, with assumed reproductive capability (i.e. female sex). Throughout this manuscript, we use the term ‘women’ to include participants coded as female in the claims databases and acknowledge that this may encompass non-cisgender individuals. Pregnant women were identified using a previously-validated algorithm that ascertained pregnancy-related procedures, pregnancy outcomes and related dates, which were used to estimate gestational age at the end of pregnancy.(12) The estimated date of last menstrual period (LMP) was derived by subtracting the gestational age at the end of pregnancy (in completed weeks) from the infant’s date of birth. Women were eligible for inclusion in the cohort if they (1) had a pregnancy overlapping the study period, (2) had prescription benefits coverage (to ensure complete capture of immunization data), and (3) were continuously enrolled in a participating healthcare plan from 12 months prior to estimated LMP through to the end of pregnancy event (Supplementary Material).

### Exposure

COVID-19 vaccine exposures during pregnancy, defined as those administered from LMP through to one day prior to the end of pregnancy, were identified from outpatient and inpatient claims and outpatient pharmacy records using National Drug codes and Current Procedural Terminology (CPT) codes (Table 1S, Supplementary Material). The date of vaccination was defined as the date of the medical encounter in which the vaccine was received. Gestational age at vaccination (in weeks) was calculated by subtracting LMP from the vaccination date and dividing by 7.

Vaccination was treated as a time-varying exposure, with person-time classified into six categories by dose number and trimester based on the date of vaccine administration: (0) unvaccinated; or vaccinated with a single dose during (1) first trimester (0–13 weeks), (2) early second trimester (14–20 weeks), (3) late second trimester (21–27 weeks), (4) third trimester (28–42 weeks), or (5) vaccinated with ≥ 2 doses during pregnancy (regardless of gestational age at the subsequent dose(s)). Among women vaccinated with ≥ 2 doses during pregnancy, the first dose was captured in earlier trimester-specific categories (categories 1 to 4), and any subsequent doses were captured once only in a final category (category 5) (Supplementary Material).

### Outcomes

The outcomes of interest were (1) any medically-attended COVID-19 outpatient or inpatient diagnosis during pregnancy (herein referred to as a COVID-19 case) and (2) COVID-19-related hospitalization during pregnancy (herein referred to as a COVID-19 hospitalization). COVID-19 cases were identified from outpatient and inpatient claims, using International Classification of Diseases, Tenth Revision, Clinical Modification (ICD-10-CM) (see Supplementary Material). COVID-19 hospitalization was defined as any inpatient admission with a corresponding COVID-19 diagnosis. Only outcomes occurring from the date of LMP through to one day prior to the end of pregnancy were captured.

### Statistical Analyses

Descriptive statistics (counts and percentages, crude incidence rates per 100,000 of person-days at risk, medians, minimum, maximum and interquartile ranges [IQR]) were used to describe participant characteristics, follow-up time and outcomes overall and by time-varying vaccination status. Cox models were used to estimate hazard ratios (HRs) for each outcome of interest comparing vaccinated with unvaccinated women. Vaccination was modeled as a time-varying exposure, with follow-up time partitioned into intervals reflecting changes in vaccination status over time. Women were considered vaccinated beginning 14 days after receipt of each COVID-19 vaccine dose to allow for the development of an immune response. Accordingly, outcomes occurring within 14 days of the first vaccine dose were attributed to unvaccinated person-time. Unvaccinated person-time commenced at LMP until change in vaccination status or end of follow-up. End of follow-up was defined as the earliest of either date of the outcome, or date of pregnancy end. Pregnancy endpoints included live birth, stillbirth, spontaneous or medical abortion, ectopic pregnancy, trophoblastic pregnancy, or pregnancy of unknown outcome.

Stabilized inverse probability treatment weights (IPTW) were used to standardize estimates for measured baseline confounders. IPTWs were winsorized at the 1st and 99th percentiles of the weight distribution to improve stability and limit the influence of extreme values.(12) Propensity scores were estimated using multinomial logistic regression models, yielding predicted probabilities for all possible exposure levels. Baseline socio-demographic and clinical covariates were maternal age, insurance provider type, smoking status (a composite of clinically-coded tobacco use complicating pregnancy, childbirth and the puerperium or clinically-coded history of smoking in the 12 months prior to LMP), drug use complicating pregnancy, childbirth and the puerperium, history of cardiovascular disease, history of respiratory disease or immunocompromising condition(s), disability, history of preterm birth, COVID-19 diagnosis prior to pregnancy, influenza vaccine prior to pregnancy, and COVID-19 vaccine prior to pregnancy (Table 2S and Table 3S, Supplementary Material).

Covariate balance for the categorical exposure was assessed by computing pairwise standardized mean differences (SMD) comparing each exposure level with the unvaccinated reference group. For each covariate, balance was summarized using the maximum absolute SMD across pairwise comparisons, with SMD < 0.10 considered indicative of adequate balance. Covariates that remained imbalanced after weighting were included as direct adjustments in the outcome models, consistent with a doubly robust analytic approach. Additional direct adjustments were made for calendar time of pregnancy (year and quarter of LMP) to account for secular trends, and weekly nationally reported COVID-19 case counts (logtransformed) to capture variation in force of infection. Details of the covariates and balance diagnostics are given in the Supplementary Material. VE was calculated as (1–HR) × 100%.

We additionally conducted stratified analyses of VE by epidemic wave. Women were classified according to the epidemic wave in which their pregnancy ended (Wildtype/Alpha: <1 June 2021; Delta: 1 June 2021–20 December 2021; Omicron > 20 December 2021). A sensitivity analysis was conducted with follow-up censored 5 days prior to the end of pregnancy event to exclude outcomes potentially captured through screening associated with birth admissions, which are likely to be non-differential by vaccination status and may include asymptomatic SARS-CoV-2 infections.

Data preparations were performed in R (version 4.4.2) and Stata 18.0 (StataCorp LLC, College Station, TX). Cox proportional hazards models were fitted using the coxph() function from the *survival* package (version 3.7–0) in R (version 4.4.2).(13)

We followed the **RE**porting of studies **C**onducted using **O**bservational **R**outinely collected health **D**ata (RECORD) Statement.(13) The study was conducted using de-identified administrative health data and was deemed by the [redacted for peer review] Institutional Review Board to be non-human subjects research and exempt from full review.

## RESULTS

**Figure 1:**
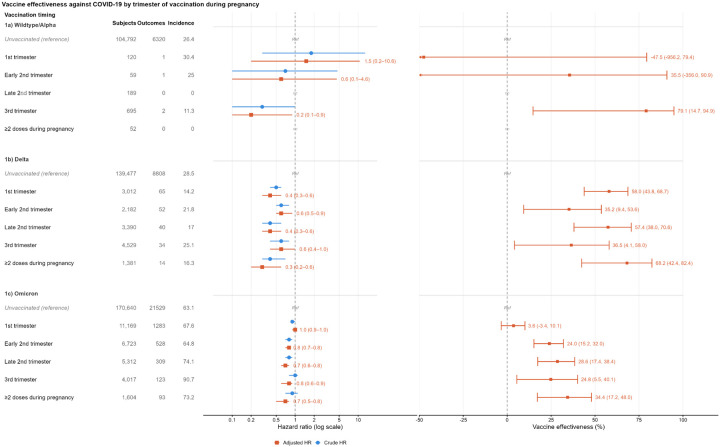
Hazard ratios, vaccine effectiveness estimates, and 95% confidence intervals (reference: unvaccinated during pregnancy) against maternal COVID-19 cases, by dose and trimester of COVID-19 vaccination during pregnancy, stratified by epidemic wave*. NOTES: * Epidemic wave defined by predominance of SARS-CoV-2 variants given the end of pregnancy event (EOPE): Wildtype/Alpha = EOPE < 1 June 2021; Delta = EOPE during Delta period or started and continued throughout delta period, defined as 1 July 2021 – 20 December 2021, inclusive; Omicron = EOPE > 20 December 2021 † Exposure status definitions: Unvaccinated = unvaccinated during the current pregnancy; first trimester = 0–13 weeks; early second trimester = 14–20 weeks; late second trimester = 21-27 weeks; third trimester = 28–42 weeks; Vaccinated with ≥2 doses during pregnancy = two or more doses of COVID-19 vaccine administrated at any time during the current pregnancy. Ref=Reference; HR=Hazard ratio; VE=Vaccine effectiveness; LCI=Lower confidence interval; UCI=Upper confidence interval; nc = not calculable. ^**‡**^ Subject and outcome counts reflect person-time contributions under time-varying vaccination exposures. Subjects may therefore be represented in multiple exposure categories if vaccination status changed during follow-up. Outcome (failure events) are attributed to the exposure level at the time of failure event occurred. Accordingly, subject and outcome counts will differ to our descriptive tables. **§** Inverse probability treatment weights (IPTW) were used to standardize estimates for measured confounders: maternal age, insurance provider type, smoking status, drug use during pregnancy, history of cardiovascular disease, respiratory disease, immunocompromising condition, disability, history of preterm birth, COVID-19 diagnosis prior to pregnancy, influenza vaccine prior to pregnancy, and COVID-19 vaccine prior to pregnancy. Additional direct adjustments were made for calendar time (quarter of the last menstrual period) and the log of weekly, nationally-reported COVID-19 cases in the US.

**Figure 2:**
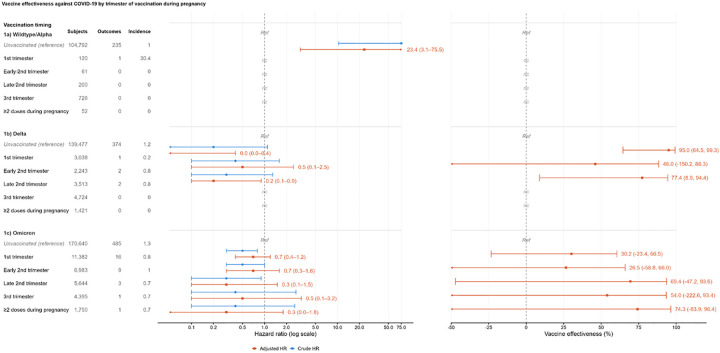
Hazard ratios, vaccine effectiveness estimates, and 95% confidence intervals (reference: unvaccinated during pregnancy) against maternal COVID-19 hospitalizations, by dose and trimester of COVID-19 vaccination during pregnancy, stratified by epidemic wave^†^. NOTES: * Subject and outcome counts reflect person-time contributions under time-varying vaccination exposures. Subjects may therefore be represented in multiple exposure categories if vaccination status changed during follow-up. Outcome (failure events) are attributed to the exposure level at the time of failure event occurred. Accordingly, subject and outcome counts will differ to our descriptive tables; Ref = Reference; HR = Hazard ratio; VE = Vaccine effectiveness; LCI = Lower confidence interval; UCI = Upper confidence interval; nc = not calculable. ^ Inverse probability treatment weights (IPTW) were used to standardize estimates for measured confounders: maternal age, insurance provider type, smoking status, drug use during pregnancy, history of cardiovascular disease, respiratory disease, immunocompromising condition, disability, history of preterm birth, COVID-19 diagnosis prior to pregnancy, influenza vaccine prior to pregnancy, and COVID-19 vaccine prior to pregnancy. Additional direct adjustments were made for calendar time (quarter of the last menstrual period) and the log of weekly, nationally-reported COVID-19 cases in the US. † Epidemic wave defined by predominance of SARS-CoV-2 variants given the end of pregnancy event (EOPE): Wildtype/Alpha = EOPE < 1 June 2021; Delta = EOPE during Delta period or started and continued throughout delta period, defined as 1 July 2021 – 20 December 2021, inclusive; Omicron = EOPE > 20 December 2021.

A total of 698,644 women were assessed for eligibility, and after exclusions, 414,909 were included in the study cohort (Fig. 1S, Supplementary Material). Characteristics of the total cohort and by COVID-19 case status are given in Table 1. Overall, 58,975 (14.2%) received ≥ 1 COVID-19 vaccine during pregnancy and 355,934 (85.8%) remained unvaccinated throughout pregnancy. Among women vaccinated, 4.0% had their vaccine administered in the first trimester, 2.9% each in the early and late second trimester, and 3.6% in the third trimester. There were 3,644 (0.9%) women who received more than two doses of vaccine during pregnancy. We identified 39,202 (9.4%) COVID-19 cases during pregnancy, of which 1,130 (n = 1,130/39,202; 2.9%) required hospitalization. Most COVID-19 cases occurred in women whose pregnancies ended during the Omicron epidemic period. Proportionally more COVID-19 cases smoked or had a record of underlying cardiovascular or respiratory complications during the 12 months prior to pregnancy. The two groups were similar across other socio-demographic characteristics, including maternal age and insurance provider type. Relative to gestational age, proportionally more COVID-19 cases occurred in the late second and early third trimester. This pattern was most notable among COVID-19 hospitalizations (Fig. 2S, Supplementary Material). After IPTW were applied, most covariates were balanced by exposure status; however, maternal age remained partially unbalanced (maximum SMD = 0.14) and was therefore additionally included in the outcome models, consistent with a doubly robust estimation approach (Table 4S, Supplementary Material).

Follow-up time was partitioned according to time-varying vaccination status, with women contributing 97,616,515 person-days at risk. Most COVID-19 cases (n = 36,657/39,202; 93.5%) occurred during unvaccinated person-time, corresponding to a crude incidence rate of 41.2 per 100,000 unvaccinated person-days. COVID-19 incidence, unadjusted HRs and adjusted HRs and VE estimates by exposure status are shown in Table 2. Overall, VE was very low following first trimester vaccination (VE = 9.6%, 95% CI: 3.2%–15.5%) and low-to-moderate across all subsequent time points. The highest VE was observed following late second trimester vaccination (VE = 36.3%, 95% CI: 26.9%–44.4%) and among women with ≥ 2 doses of vaccine administered at any time during pregnancy (VE = 40.7%, 95% CI: 26.3%–52.3%), corresponding to a crude incidence rate of 52.6 and 49.8 per 100,000 person-days, respectively. The VE estimates showed a general pattern of increasing protection during later gestational periods or when ≥ 2 doses were administered. This trend did not persist following third trimester vaccination (VE = 29.7%, 95% CI: 14.1%–42.4%). However, in a sensitivity analysis where follow-up was censored 5 days prior to the end of pregnancy event, this attenuation was no longer observed, with VE following third trimester vaccination increasing by 6 percentage points to 35.3% (95% CI: 18.8%–48.4%) (Table 5S, Supplementary Material).

COVID-19 incidence and VE estimates varied by epidemic wave (Fig. 1). There were 6,324 COVID-19 cases identified during the Wild-type/Alpha epidemic period (26.4 cases per 100,000 person-days), with all but four cases occurring during unvaccinated person-days. There were 9,013 COVID-19 cases identified during the Delta epidemic period (28.1 cases per 100,000 person-days), with moderate-to-good protection observed following one or more doses administered during pregnancy across all trimesters. Among women vaccinated with one dose during the period of Delta predominance, VE ranged between 35.2% (95% CI: 9.4%–53.6%) following early second trimester vaccination to 58.0% (95% CI: 43.8%–68.7%) following first trimester vaccination. VE was highest among women with ≥ 2 doses of vaccine administered any time during pregnancy (16.3 cases per 100,000 person-days; VE = 68.2%, 95% CI: 42.4%–82.4%). Overall, a higher incidence of COVID-19 cases in women was observed during the period of Omicron predominance (63.7 cases per 10,000 person-days), but generally lower VE estimates compared to those observed in the period of Delta predominance (Fig. 1).

In our analysis estimating VE against COVID-19 hospitalization, most hospitalizations (n = 1,094/1,130; 96.8%) occurred during unvaccinated person-time, corresponding to a crude incidence rate of 1.2 per 100,000 unvaccinated person-days (Table 3). Although numbers were sparse and confidence intervals wide, the VE point estimates increased by dose and trimester of vaccination. VE was modest following first trimester and early second trimester vaccination but increased thereafter. Late second trimester vaccination conferred good protection (0.7 cases per 100,000 person-days; VE = 73.1%, 95% CI: 13.3%– 91.6%), with similar protection observed following third trimester vaccination (0.3 cases per 100,000 person-days; VE = 75.6%, 95% CI: −72.0%–96.5%). The point estimate was highest following ≥ 2 doses of vaccine administered any time during pregnancy (0.4 cases per 100,000 person-days; VE = 81.5%, 95% CI: −31.7%–97.4%) (Table 3). Sparse outcomes precluded reliable estimation of VE against COVID-19 hospitalization when stratified by the Wild-type/Alpha epidemic wave, with most strata containing zero events. Although numbers were similarly small during the period of Delta predominance, we observed very high VE following first trimester vaccination (VE = 95.0%, 95% CI: 64.5%–99.3%) and moderate VE following late second trimester vaccination (VE = 77.4%, 95% CI: 8.9%–94.4%). During Omicron predominance, we observed low-to-moderate VE across all trimesters with the highest following late second trimester vaccination (0.7 cases per 100,000 person-days; VE = 69.4%, 95% CI: −47.1%–93.6%), or ≥ 2 doses of vaccine administered any time during pregnancy (0.7 cases per 100,000 person-days; VE = 74.3%, 95% CI:−83.9%–96.4%) (Fig. 2).

## DISCUSSION

In this large, claims-based cohort study, we found that COVID-19 vaccination during pregnancy conferred good protection against severe maternal COVID-19 requiring hospitalization. Although VE estimates were imprecise due to small numbers, with estimates in some strata statistically non-significant, point estimates tended to increase when vaccination occurred later in gestation or when ≥ 2 doses were administered at any time during pregnancy (ranging from 73.1% following late second trimester vaccination through to 81.5% following administration of ≥ 2 doses during pregnancy). We observed low-to-moderate protection against the less severe endpoint (COVID-19 case), with similarly higher VE during later periods of gestation or following additional doses. Although other studies have demonstrated the effectiveness of COVID-19 vaccines when administered at any time during pregnancy; the trimester of vaccine exposures is poorly reported (4) precluding trimester-specific VE estimates. Our study addresses this evidence gap by providing real-world evidence to inform patient, provider, and policy-maker decision-making aimed at optimizing protection against maternal COVID-19.

Our low-to-moderate VE estimates against medically-attended disease are consistent with the literature – in both general (14) and pregnant (4, 15) populations – showing moderate VE for less severe and/or non-specific outcomes following one dose of vaccine. In our study, outcomes were ascertained from ICD-10-coded COVID-19 diagnoses, which included clinically-diagnosed outcomes without laboratory confirmation across both outpatient and inpatient settings during a period of heightened testing for COVID-19. This likely captured some asymptomatic or mildly-symptomatic infections, attenuating VE relative to more severe and specific endpoints.(16) Additionally, these estimates were derived from our total cohort, spanning the Wild-type/Alpha, Delta and Omicron epidemic waves in the US, yielding an overall VE estimate across periods with known heterogeneity. Relative to other epidemic periods, VE was generally higher during the period of Delta predominance (ranging from 35.2% following early second trimester vaccination to 68.2% following ≥ 2 doses during pregnancy). This aligns with the existing literature, which demonstrates higher VE during periods of Delta predominance in pregnant populations.(17, 18) In our analysis examining COVID-19 hospitalizations, VE was good following late second (73.1%) or third (75.6%) trimester vaccination. These findings are consistent with prior studies demonstrating strong effectiveness of COVID-19 vaccination during pregnancy against severe maternal outcomes.

The higher VE observed against both COVID-19 cases (40.7%) and COVID-19 hospitalizations (81.5%) among women who received ≥ 2 doses of vaccine during pregnancy likely reflects protection associated with completion of the two-dose primary COVID-19 vaccination series, as was recommended by manufacturers during the COVID-19 vaccine rollout.(19, 20) Completion of a two-dose vaccination series is particularly important during the emergence of a new pathogen to which the population is not previously immune and for which a prime-boost regimen is appropriate to achieve protective immunity. Most women in our study were vaccine-naïve, with only 10.2% having received a COVID-19 vaccine prior to pregnancy and fewer than 6% having a pre-pregnancy COVID-19 diagnosis. Importantly, both COVID-19 vaccination and diagnoses occurring in the 12 months prior to pregnancy were explicitly accounted for in our IPTWs, suggesting that the observed differences reflect protection from vaccination during pregnancy, rather than differences in baseline vaccine- or infection-induced immunity.

In our analysis of COVID-19 hospitalizations, we observed higher VE during later periods of gestation and following administration of additional doses. For our analysis examining COVID-19 cases, this pattern was less obvious, with variable VE across all trimesters, and a modest attenuation of VE following third-trimester vaccination, despite an otherwise increasing gradient of protection across all other periods of gestation. This is likely an artefact reflecting increased healthcare contact and routine screening around admission for delivery, whereby both vaccinated and unvaccinated women are equally likely to be tested and have an infection detected, including asymptomatic or mildly symptomatic cases. This contrasts from testing earlier in pregnancy, which is more likely to be symptom-driven. This form of ascertainment bias disproportionately affects vaccine exposures occurring closer to term and is a known challenge in pregnancy studies.(21) In a sensitivity analysis censoring follow-up five days prior to the end of pregnancy, this attenuation was no longer observed, and VE estimates more consistently followed a modest pattern of increasing protection during later periods of gestation and additional doses. Taken together, these findings suggest that vaccination during later periods of gestation may provide moderate or good protection against maternal COVID-19 cases or hospitalization, respectively, when the risk of severe maternal disease is highest.(22)

Very few studies have estimated VE against maternal disease by trimester of vaccination. One currently unpublished target trial emulation using data from the United Kingdom, Sweden, Spain and Norway reported low VE estimates against both clinically-diagnosed or laboratory-confirmed COVID-19 and COVID-19-related hospitalization, noting no variation in VE by trimester.(23) Study design differences limit comparability, including limited follow-up to observe outcomes (median 44–55 days), and their trimester-specific estimates spanning pandemic and post-pandemic periods where variants were not well-matched to the vaccine. Our findings are, however, consistent with a growing body of immunogenicity and real-world effectiveness studies demonstrating that the timing of vaccination during pregnancy has implications for both maternal and infant protection. Immunogenicity studies consistently show higher neutralizing antibody concentrations in maternal and cord blood,(10) as well as more durable antibody persistence in infants (24, 25) when vaccination is administered later in gestation particularly in the third trimester, lending biological plausibility to our observed estimates. In contrast, vaccination earlier in gestation is associated with greater antibody waning by the time of delivery.(26) Complementing these mechanistic data, population-based studies have demonstrated meaningful VE against both symptomatic and severe COVID-19 in pregnant women, along with evidence of antibody waning when vaccination precedes pregnancy by several months.(27) Furthermore, a recent study has demonstrated higher VE against infant disease when women are vaccinated in the third trimester.(8) Together, these findings provide biological and epidemiologic context for patterns we observed in our study, while reinforcing that vaccination at any point during pregnancy confers protection against maternal COVID-19, with additional downstream benefits for infants when vaccination occurs later in gestation.

Regardless of these trimester-specific results, the most striking observation is that most COVID-19 cases and hospitalizations occurred during unvaccinated person-time. This underscores the ongoing need for vaccination during pregnancy among those who remain unvaccinated or under-vaccinated. Although the ACOG and Infectious Diseases Society of America continue to recommend COVID-19 vaccination during pregnancy, the policy landscape in the US and elsewhere remains fragmented. The US Centers for Disease Control and Prevention (CDC) and the Advisory Committee on Immunization Practices (ACIP) have shifted away from a universal recommendation for COVID-19 vaccination during pregnancy, instead centering guidance on individual decision-making.(28) Although the CDC acknowledges that COVID-19 vaccination provides the *‘greatest benefit if you are at higher risk for severe illness, including if you are pregnant’*, the absence of a universal recommendation is likely to contribute to further declines in vaccination uptake in pregnancy, a group for whom coverage has been,(29) and continues to be,(30) suboptimal, with recent estimates indicating that only 11% of women are vaccinated during pregnancy.

Our study’s strengths include the use of national data capturing a large cohort of pregnancies and inclusion of socioeconomically diverse women such as Medicaid beneficiaries, enhancing the transportability of findings. Nonetheless, several limitations should be noted. First, misclassification of both COVID-19 vaccination and diagnosis during pregnancy is possible, as exposures and outcomes were calculated based on the estimated date of LMP. If LMP was estimated with error, this may have inadvertently led to misclassification of these variables relative to gestational time. However, we used a validated algorithm to estimate LMP, which has high agreement against physician-adjudicated electronic health records and is widely used in post-marketing maternal vaccine safety surveillance.(31) Second, COVID-19 cases and hospitalizations were ascertained from claims-based outpatient or inpatient records, and women without a claim were classified as outcome-free. Given the widespread circulation of SARS-CoV-2 during the study period, some women may have experienced asymptomatic or minimally symptomatic infections not prompting medical care. These infection nus would not be captured in claims data, potentially resulting in outcome misclassification. Accordingly, our outcome definitions were limited to medically-attended events clinically coded as COVID-19, and findings may not be generalizable to all SARS-CoV-2 infections nor to medically-attended events not recognized as COVID-19. Third, we were unable to fully disentangle the effects of gestational timing of vaccination from concurrent epidemic activity. Although we adjusted for calendar time of pregnancy and weekly nationally-reported COVID-19 case counts to account for temporal trends and variations in force of infection, we could not jointly stratify or model VE by both gestational timing and epidemic intensity. As such, some residual confounding by epidemic context cannot be excluded. Fourth, despite the large cohort size, COVID-19 hospitalizations were uncommon leading to imprecise trimester-specific estimates. This underscores the need for very large, pooled, or multinational studies to more precisely estimate VE against rarer maternal outcomes. Furthermore, we were unable to distinguish whether hospitalizations occurred due to COVID-19 or whether COVID-19 was an incidental diagnosis accompanying an alternative primary cause of admission. Fifth, although our use of IPTWs incorporated several important confounders – including insurance provider type, smoking status and other medical comorbidities seldom reported in other studies – residual or unmeasured confounding could persist. Finally, misclassification of vaccination, outcome and confounder status is possible due to data linkage errors (32) and missing data (33).

Our study provides evidence that COVID-19 vaccination at any point during pregnancy confers clinically-meaningful protection against maternal COVID-19, particularly severe disease requiring hospitalization, with point estimates suggesting greater protection when vaccination occurs during later gestational periods or after receipt of additional doses. In the context of reduced COVID-19 incidence and evolving public health recommendations, these trimester-specific estimates add important information for patients, clinicians and policymakers seeking to optimize protection during pregnancy. Although further studies are needed to evaluate the impact of maternal vaccination on infant COVID-19 outcomes, our results address a key evidence-gap relating to timing of vaccination during pregnancy to maximize protection against medically-attended maternal COVID-19. Notably, most COVID-19 cases and hospitalizations occurred during unvaccinated person-time, underscoring that the primary clinical and public health priority remains ensuring that pregnant women who remain un- or under-vaccinated receive COVID-19 vaccination during pregnancy, regardless of gestational timing.

## Supplementary Material

Supplementary Files

This is a list of supplementary files associated with this preprint. Click to download.

- ROWESUPPLEMENARYMATERIALSubmitted.docx

## Figures and Tables

**Table 1:** Characteristics of the study cohort, overall and stratified by COVID-19 case status.

	COVID-19 case		
	Total	No	Yes
	N = 414,909	N = 375,707 (90.6%)	N = 39,202 (9.4%)
COVID-19 vaccination during pregnancy*			
Unvaccinated during pregnancy	355,934 (85.8%)	321,870 (85.7%)	34,064 (86.9%)
Vaccinated trimester 1	16,444 (4.0%)	14,739 (3.9%)	1,705 (4.3%)
Vaccinated early trimester 2	12,026 (2.9%)	10,980 (2.9%)	1,046 (2.7%)
Vaccinated late trimester 2	11,934 (2.9%)	10,984 (2.9%)	950 (2.4%)
Vaccinated trimester 3	14,927 (3.6%)	13,815 (3.7%)	1,112 (2.8%)
Vaccinated > = 2 during pregnancy (any time)	3,644 (0.9%)	3,319 (0.9%)	325 (0.8%)
Maternal age	30.0 (25.0–34.0)	30.0 (25.0–34.0)	30.0 (25.0–34.0)
Insurance provider type			
Private (commercial)	224,889 (54.2%)	203,853 (54.3%)	21,036 (53.7%)
Public (Medicaid)	190,020 (45.8%)	171,854 (45.7%)	18,166 (46.3%)
Gestational age at pregnancy end	38.0 (34.0–38.0)	38.0 (34.0–38.0)	38.0 (36.0–38.0)
Epidemic period at pregnancy end^†^			
Wildtype/alpha	104,792 (25.3%)	98,468 (26.2%)	6,324 (16.1%)
Delta	139,477 (33.6%)	130,464 (34.7%)	9,013 (23.0%)
Omicron	170,640 (41.1%)	146,775 (39.1%)	23,865 (60.9%)
Past or current smoker			
No	346,929 (83.6%)	315,033 (83.9%)	31,896 (81.4%)
Yes	67,980 (16.4%)	60,674 (16.1%)	7,306 (18.6%)
Recorded drug use during pregnancy			
No	390,516 (94.1%)	353,687 (94.1%)	36,829 (93.9%)
Yes	24,393 (5.9%)	22,020 (5.9%)	2,373 (6.1%)
Cardiovascular complications prior to pregnancy			
No	382,172 (92.1%)	346,655 (92.3%)	35,517 (90.6%)
Yes	32,737 (7.9%)	29,052 (7.7%)	3,685 (9.4%)
Respiratory complications prior to pregnancy			
No	331,662 (79.9%)	302,121 (80.4%)	29,541 (75.4%)
Yes	83,247 (20.1%)	73,586 (19.6%)	9,661 (24.6%)
Immunocompromising conditions prior to pregnancy			
No	409,668 (98.7%)	371,147 (98.8%)	38,521 (98.3%)
Yes	5,241 (1.3%)	4,560 (1.2%)	681 (1.7%)
Disability			
No	397,515 (95.8%)	360,312 (95.9%)	37,203 (94.9%)
Yes	17,394 (4.2%)	15,395 (4.1%)	1,999 (5.1%)
Current pregnancy a result of assisted reproductive technology			
No	405,791 (97.8%)	367,273 (97.8%)	38,518 (98.3%)
Yes	9,118 (2.2%)	8,434 (2.2%)	684 (1.7%)
Past pregnancy ending with preterm birth						
No	394,269 (95.0%)	357,300 (95.1%)	36,969 (94.3%)
Yes	20,640 (5.0%)	18,407 (4.9%)	2,233 (5.7%)
Pre-pregnancy COVID-19 diagnoses						
No	390,929 (94.2%)	354,438 (94.3%)	36,491 (93.1%)
Yes	23,980 (5.8%)	21,269 (5.7%)	2,711 (6.9%)
Influenza vaccination prior to pregnancy						
No	374,105 (90.2%)	339,296 (90.3%)	34,809 (88.8%)
Yes	40,804 (9.8%)	36,411 (9.7%)	4,393 (11.2%)
COVID-19 vaccination prior to pregnancy						
No	372,590 (89.8%)	338,083 (90.0%)	34,507 (88.0%)
Yes	42,319 (10.2%)	37,624 (10.0%)	4,695 (12.0%)

NOTES:

* Unvaccinated = unvaccinated during the current pregnancy; first trimester = 0–13 weeks; early second trimester = 14–20 weeks; late second trimester = 21–27 weeks; third trimester = 28–42 weeks; Vaccinated with ≥ 2 doses during pregnancy = two or more doses of COVID-19 vaccine administrated at any time during the current pregnancy.

† Epidemic wave defined by predominance of SARS-CoV-2 variants given the end of pregnancy event (EOPE): Wildtype/Alpha = EOPE < 1 June 2021; Delta = EOPE during Delta period or started and continued throughout delta period, defined as 1 July 2021–20 December 2021, inclusive; Omicron = EOPE > 20 December 2021

**Table 2:** Hazard ratios, vaccine effectiveness estimates, and 95% confidence intervals (reference: unvaccinated during pregnancy) against maternal COVID-19 cases, by dose and trimester of COVID-19 vaccination during pregnancy.

Exposure status^†^					Unadjusted	Adjusted^‡^	
	Subjects*	Outcomes*	Time at risk (days)	Incidence (per 100,000 person days)	HR (LCI, UCI)	HR (LCI, UCI)	VE (LCI, UCI)
Total	414,909	39,202	93,571,994	41.9			
Unvaccinated (reference)	414,909	36,657	88,987,001	41.2	Ref	Ref	Ref
Vaccinated first trimester	14,301	1,349	2,360,406	57.2	1.2 (1.2, 1.3)	0.9 (0.8, 1.0)	9.6 (3.2, 15.5)
Vaccinated early second trimester	8,964	581	1,057,824	54.9	1.1 (1.0, 1.1)	0.7 (0.7, 0.8)	26.0 (17.8, 33.4)
Vaccinated late second trimester	8,891	349	663,455	52.6	0.9 (0.8, 1.0)	0.6 (0.6, 0.7)	36.3 (26.9, 44.4)
Vaccinated third trimester	9,241	159	288,564	55.1	0.9 (0.8, 1.0)	0.7 (0.6, 0.9)	29.7 (14.1, 42.4)
Vaccinated ≥ 2 during pregnancy	3,037	107	214,744	49.8	0.9 (0.8, 1.1)	0.6 (0.5, 0.7)	40.7 (26.3, 52.3)

NOTES:

* Subject and outcome counts reflect person-time contributions under time-varying vaccination exposures. Subjects may therefore be represented in multiple exposure categories if vaccination status changed during follow-up. Outcome (failure events) are attributed to the exposure level at the time of failure event occurred. Accordingly, subject and outcome counts will differ to our descriptive tables.

† Exposure status definitions: Unvaccinated = unvaccinated during the current pregnancy; first trimester = 0–13 weeks; early second trimester = 14–20 weeks; late second trimester = 21–27 weeks; third trimester = 28–42 weeks; Vaccinated with ≥ 2 doses during pregnancy = two or more doses of COVID-19 vaccine administrated at any time during the current pregnancy. Ref = Reference; HR = Hazard ratio; VE = Vaccine effectiveness; LCI = Lower confidence interval; UCI = Upper confidence interval.

‡ Inverse probability treatment weights (IPTW) were used to standardize estimates for measured confounders: maternal age, insurance provider type, smoking status, drug use during pregnancy, history of cardiovascular disease, respiratory disease, immunocompromising condition, disability, history of preterm birth, COVID-19 diagnosis prior to pregnancy, influenza vaccine prior to pregnancy, and COVID19 vaccine prior to pregnancy. Additional direct adjustments were made for calendar time (quarter of the last menstrual period) and the log of weekly, nationally-reported COVID-19 cases in the US.

**Table 3:** Hazard ratios, vaccine effectiveness estimates, and 95% confidence intervals (reference: unvaccinated during pregnancy) against maternal COVID-19 hospitalization, by dose and trimester of COVID-19 vaccination during pregnancy.

Exposure status*					Unadjusted	Adjusted^†^	
	Subjects	Outcomes	Time at risk (days)	Incidence (per 100,000 person days)	HR (LCI, UCI)	HR (LCI, UCI)	VE (LCI, UCI)
Total	414,909	1,130	97,616,515	1.2			
Unvaccinated (reference)	414,909	1,094	92,735,041	1.2	Ref	Ref	Ref
Vaccinated first trimester	14,540	18	2,503,967	0.7	0.5 (0.3, 0.8)	0.6 (0.3, 1.0)	41.1 (−1.0, 65.6)
Vaccinated early second trimester	9,287	11	1,125,625	1.0	0.5 (0.3, 0.9)	0.7 (0.3, 1.3)	32.6 (−34.2, 66.1)
Vaccinated late second trimester	9,357	5	710,756	0.7	0.3 (0.1, 0.8)	0.3 (0.1, 0.9)	73.1 (13.3, 91.6)
Vaccinated third trimester	9,845	1	308,976	0.3	0.2 (0.0, 1.3)	0.2 (0.0, 1.7)	75.6 (−72.0, 96.5)
Vaccinated ≥ 2 during pregnancy	3,223	1	232,150	0.4	0.2 (0.0, 1.7)	0.2 (0.0, 1.3)	81.5 (−31.7, 97.4)

NOTES:

* Exposure status definitions: Unvaccinated = unvaccinated during the current pregnancy; first trimester = 0–13 weeks; early second trimester = 14–20 weeks; late second trimester = 21–27 weeks; third trimester = 28–42 weeks; Vaccinated with ≥ 2 doses during pregnancy = two or more doses of COVID-19 vaccine administrated at any time during the current pregnancy. Ref=Reference; HR=Hazard ratio; VE=Vaccine effectiveness; LCI=Lower confidence interval; UCI=Upper confidence interval.

† Inverse probability treatment weights (IPTW) were used to standardize estimates for measured confounders: maternal age, insurance provider type, smoking status, drug use during pregnancy, history of cardiovascular disease, respiratory disease, immunocompromising condition, disability, history of preterm birth, COVID-19 diagnosis prior to pregnancy, influenza vaccine prior to pregnancy, and COVID19 vaccine prior to pregnancy. Additional direct adjustments were made for calendar time (quarter of the last menstrual period) and the log of weekly, nationally-reported COVID-19 cases in the US.

## Data Availability

The data that support the findings of this study are available from Merative^™^ Marketscan^®^, however, restrictions apply to the availability of these data. These data were used under licence for the current study and are not publicly available.
